# Divergent Transcriptomic Effects of Allopregnanolone in Postpartum Depression

**DOI:** 10.3390/genes14061234

**Published:** 2023-06-08

**Authors:** Sarah A. Rudzinskas, Maria A. Mazzu, Crystal Edler Schiller, Samantha Meltzer-Brody, David R. Rubinow, Peter J. Schmidt, David Goldman

**Affiliations:** 1 Behavioral Endocrinology Branch, National Institute of Mental Health (NIMH), NIH, 10 Center Drive MSC 1277, Bethesda, MD 20892, USA; sarah.rudzinskas@nih.gov (S.A.R.);; 2 Laboratory of Neurogenetics, National Institute on Alcohol Abuse and Alcoholism (NIAAA), NIH, Rockville, MD 20855, USA; 3 Department of Psychiatry, University of North Carolina, Chapel Hill, NC 27599, USA

**Keywords:** postpartum depression, allopregnanolone, transcriptomics, neurosteroids, *GAD1*

## Abstract

Brexanolone, a formulation of the neurosteroid allopregnanolone (ALLO), is approved for treating postpartum depression (PPD) and is being investigated for therapeutic efficacy across numerous neuropsychiatric disorders. Given ALLO’s beneficial effects on mood in women with PPD compared to healthy control women, we sought to characterize and compare the cellular response to ALLO in women with (*n* = 9) or without (*n* = 10, i.e., Controls) past PPD, utilizing our previously established patient-derived lymphoblastoid cell lines (LCLs). To mimic in vivo PPD ALLO-treatment, LCLs were exposed to ALLO or DMSO vehicle for 60 h and RNA-sequenced to detect differentially expressed genes (DEGs, p_nominal_ < 0.05). Between ALLO-treated Control and PPD LCLs, 269 DEGs were identified, including Glutamate Decarboxylase 1 (*GAD1*), which was decreased 2-fold in PPD. Network analysis of PPD:ALLO DEGs revealed enriched terms related to synaptic activity and cholesterol biosynthesis. Within-diagnosis analyses (i.e., DMSO vs. ALLO) detected 265 ALLO-induced DEGs in Control LCLs compared to only 98 within PPD LCLs, with just 11 DEGs overlapping. Likewise, the gene ontologies underlying ALLO-induced DEGs in PPD and Control LCLs were divergent. These data suggest that ALLO may activate unique and opposing molecular pathways in women with PPD, which may be tied to its antidepressant mechanism.

## 1. Introduction

The FDA approval of brexanolone critically advanced the treatment of postpartum depression (PPD), a highly prevalent [1,2], clinically distinct mood disorder with a myriad of negative health consequences [3,4,5]. Brexanolone is the proprietary, chemical formulation of allopregnanolone (ALLO), a neurosteroid metabolite of progesterone (P4) and positive allosteric modulator of the GABA_A_ receptor (GABA_A_R) [6]. Clinical studies [7,8] of brexanolone for PPD were supported by pre-clinical data demonstrating that ALLO-induced, GABA_A_R-mediated synaptic activity can promote both anxiolytic- [9] and antidepressant- [10,11] like effects in rodents as well as modulate maternal care and stress in transgenic mouse models for PPD [12,13]. Although ALLO’s cellular mechanisms and molecular consequences in PPD have yet to be fully understood [14,15], numerous studies have or are currently investigating ALLO’s potential to treat a vast range of neuropsychiatric conditions from major depressive disorder [16] to Alzheimer’s disease [17].

We previously hypothesized that women with PPD have an innate cellular sensitivity to changes in ovarian steroids that underlies their affective symptoms [18]. Recent findings from our patient-derived, lymphoblastoid cell lines (LCLs) support this hypothesis, demonstrating intrinsically different transcriptomes in PPD LCLs compared to controls, both in the absence of exogenous hormonal variation (baseline) and in response to the addition and removal of estradiol (E2) and P4 [19]. These in vitro data provide a possible biological basis for the clinical observation that the recapitulation of ovarian steroid changes occurring peripartum can trigger a recurrence of mood symptoms in women with past PPD but not matched postpartum controls [20,21,22].

Whether women with PPD have a similar differential mood response to ALLO has yet to be clinically determined. Unlike E2 and P4, where endogenous levels do not differ in women with PPD compared with controls, evidence regarding whether this holds true for ALLO is mixed [23,24,25]. In healthy control women, certain [26] exogenous doses of ALLO can induce fatigue [27,28] and memory deficits [29], and ALLO may ultimately be responsible for the documented sedative effects following P4 [30] or pregnanolone [31] administration. In further contrast to the mood-enhancing effects experienced by women with PPD, women with premenstrual dysphoric disorder (PMDD) experience negative mood symptoms during luteal phase increases in ALLO, with this effect blocked by dutasteride (an inhibitor of 5α-reductase, the rate limiting enzyme for ALLO production) [32,33,34]. Taken together, these clinical observations suggest that even amongst reproductive-related mood disorders, ALLO’s effects on mood and behavior could depend upon innate biological differences.

Therefore, our in vitro findings [19] of intrinsic transcriptomic differences in PPD LCLs raise questions about ALLO’s genomic effects. Namely, what are the downstream cellular responses to ALLO, and do these responses differ for, or mitigate differences in, the PPD transcriptome? LCLs are ovarian steroid-responsive [35] and have a functional GABAergic system [36,37,38]. Post-GABAR-mediated responses to ALLO could alter the expression of any number of downstream molecular players, possibly reflecting gene networks involved in PPD symptom onset or remission [39,40]. Such effects could also occur through other ALLO-responsive receptors, including neurotransmitter and immune toll-like [41,42], pregnane X [43,44], or membrane progesterone [45,46] receptors, all of which can trigger intracellular actions leading to alterations in gene regulation and expression.

To investigate these questions, here we subject our previously described [19] PPD LCL model to 60 h of ALLO-treatment and utilize transcriptomics to characterize and compare gene expression thereafter. Our goal was to identify ALLO-related transcriptomic changes (or lack thereof) in both PPD and control LCLs that could potentially be involved in ALLO’s antidepressant mechanisms for PPD.

## 2. Methods

### 2.1. Lymphoblastoid Cell Lines

Participants were euthymic women with a history of past PPD and matched postpartum controls with no history of PPD or other Axis 1 disorder recruited at the University of North Carolina, Chapel Hill (UNC). All women were interviewed to assess medical and psychiatric history and screened with physical and gynecological exams, confirming that each was otherwise healthy. Participants then underwent a clinical study at UNC, described in detail in Schiller et al., 2022 [22], characterizing their behavioral and affective response to supraphysiologic ovarian steroids. The NIH and UNC IRBs approved the study protocol, and all women provided written informed consent. At least a year after the conclusion of the clinical study, participants were re-contacted and re-consented to provide a new blood sample that was used to generate lymphoblastoid cell lines (LCLs).

### 2.2. Experimental Treatment Paradigm

**Figure 1:**
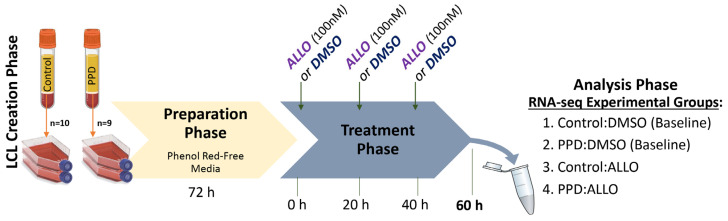
**LCL Allopregnanolone treatment paradigm/experimental design**. Blood samples were transformed using Epstein Barr Virus (EBV) to generate patient-derived lymphoblastoid cell lines (LCLs). After exponential growth and passaging (Creation Phase), LCLs (N = 19) were transferred to steroid-free media (phenol red-free, KOSR-supplemented RPMI) divided into two flasks (i.e., one per treatment) seeded at approximately 1 × 10^6^ cells/mL (Preparation Phase). Experimental treatments (baseline/vehicle (“DMSO”) and allopregnanolone (“ALLO”)) for each LCL occurred simultaneously, with either DMSO or ALLO (100 nM) given every 20 h over the course of 60 h total (Treatment Phase). Thus, four experimental groups were generated upon LCL collection via pelleting for downstream assays and Ampliseq transcriptomic analysis (Analysis Phase). Figure created in Biorender.com.

Epstein-Barr Virus generation, passaging and maintenance for LCLs prior to the experimental ALLO treatments are as described in Rudzinskas et al., 2023 [19]. For the present study, *N* = 19 LCLs were utilized, comprising *n* = 10 derived from women without a history of PPD (i.e., Controls) and *n* = 9 from women with past PPD (i.e., PPD). Approximately three days prior to experimental treatments, each LCL was seeded at ~1 × 10^6^ cells/mL in two flasks of 15%-knockout serum replacement (KOSR) RPMI-1640 (a phenol red-free [47], FBS-free [48] media). Vehicle treatment stock comprised of 100 μL sterile DMSO (Sigma-Aldrich, St. Louis, MO, USA, D2650-5X5ML) in 20 mL of media. For ALLO treatment stock, 5 mg of allopregnanolone (Sigma-Aldrich, USA, P8887-5MG) was dissolved in 1 mL of DMSO, with 100 μL added to 20 mL of media. The experimental timeline thereafter is described and depicted in Figure 1. In summary, LCLs were treated every 20 h over a 60-h period (mimicking the length of brexanolone infusion for PPD) with either an acute 100 nM spike of allopregnanolone (ALLO) or vehicle (DMSO), totaling three spikes [300 nM total] throughout the experimental treatment. Each LCL flask (*N* = 38) was then pelleted, frozen and stored at −80 °C for subsequent molecular analyses.

### 2.3. AmpliSeq-RNA Transcriptomics

TRIzol-isolated LCL pellets were RNA extracted using the Qiagen RNeasy Mini Kit (Qiagen, Germantown, MD, USA, #15596018) with RNase-free DNase (Qiagen, #79254), according to manufacturer’s instructions. Total RNA quantity and quality were assessed on the nanodrop, 100 ng of which was then used as input into the SuperScript^®^ VILO™ cDNA Synthesis kit. Total cDNA output was used according to manufacturer protocol in the AmpliSeq™ Transcriptome Human Gene Expression Kit (ThermoFisher, Waltham, MA, USA, #A26326). Final amplicon library quality and concentration was assessed using the Agilent^®^ 2100 Bioanalyzer High Sensitivity DNA Kit (Agilent, Santa Clara, CA, USA, #5067-4626), according to manufacturer’s directions.

Barcoded, prepared cDNA AmpliSeq libraries were then sequenced on the Ion Torrent S5 Sequencing System via the Ion Torrent 540-OT2 kit (ThermoFisher, USA, #A27753), using the Ion 540™ Chip Kit (ThermoFisher, USA, #A27766). Approximately eight samples were loaded in equal concentrations per chip. In contrast to whole-transcriptome RNA-seq, AmpliSeq utilizes targeted amplification via a pre-determined primer pool of up to 20k transcript targets (some ~10k of which are thought to be expressed in LCLs) to generate short amplicon tags of ~150 bps that are amplified and sequenced. Since each single read equates to the expression of a transcript (regardless of factors, such as transcript length, etc.), only ~1 million reads/sample are necessary to accurately quantify gene expression. Our libraries were sequenced at a minimum depth of 4 million high-quality reads, with an average of 8 million counts/sample. Raw AmpliSeq data was processed within the Ion Torrent sequencer algorithm, using the Torrent Mapping Alignment Program (TMAP), SamTools and the AmpliSeqRNA plugin. Together, these plugins generated matrices containing total counts (i.e., tags),per million normalized (CPM) counts of the targeted genes, and “CHP” files (binary storage of the above values in endian format for processing).

### 2.4. RNA-Seq Data Analysis with TAC

CHP files for all samples (*n* = 38) were imported into the Transcriptome Analysis Console Software (TAC) 4.0.2, along with corresponding metadata for sequencing batches and two user-defined comparison groups, Diagnosis (Control or PPD) and Treatment (DMSO or ALLO). Differentially expressed genes (DEGs) were called using the eBayes ANOVA Method with the following settings: Array Type: hg19_AmpliSeq_Transcriptome_21K_v1_Array; Fold Change (FC): <−1.25 or >1.25; *p*-value < 0.05, FDR *p*-value < 0.1. Principal Component Analysis (PCA) examined sample level variance using default TAC settings (5000 points, distributed). Lists of normalized (log_2_) expression, Gene-Level FC and FDR/nominal Gene-Level p-values were generated for the following between-subject comparisons: Control:DMSO vs. PPD:DMSO (i.e., baseline), Control:ALLO vs. PPD:ALLO; and within-subject comparisons: Control:DMSO vs. Control:ALLO, PPD:DMSO vs. PPD:ALLO. TAC software was used to perform experiment quality control and generate AmpliSeq-related plots/sample signal graphs.

### 2.5. qRT-PCR

Confirmatory quantitative real time PCR (qRT-PCR) analysis was performed on individual DEGs of interest (defined as genes with a *p* < 0.001 nominal significance, ≥|2|-fold change between diagnoses and/or functional relevance to PPD/ALLO). cDNA was synthesized from 1 μg total RNA using the TaqMan^®^ RNA Reverse Transcription Kit (Applied Biosystems, USA, #4304134), according to manufacturer’s directions: 1 μL of a 1:10 dilution of synthesized stock cDNA was combined with 10 μL of TaqMan^®^ Universal PCR Master Mix (Applied Biosystems, Waltham, MA, USA, #4324018), 8 μL of H_2_O, and 1 μL of TaqMan Primer, including (ThermoFisher, USA, #4331182): *GAD1* [HS02512069_s1] and *ACTB* [HS99999903_m1]. Each sample was analyzed in triplicate with expression quantitated according to the recommended conditions for the 7900HT Fast Real-Time PCR System (ABI, CA, USA). Relative gene expression levels were calculated using the ΔCT value between *ACTB* (beta-Actin) expression for normalization. GraphPad Prism 9 (GraphPad, Inc., Boston, MA, USA) generated related graphs and statistical analyses, including two-way ANOVAs and Student’s *t*-tests.

### 2.6. Gene Network and Ontology Analyses

All DEGs with nominal (*p* < 0.05) significance and |FC| > 1.25 were utilized for gene network analyses via Enrichr [49], a web tool comparing user data with multiple annotated gene sets, databases and pathways; or MSigDB [50], a database for identifying overrepresented molecular functions and ontologies in gene sets. Visual output was generated via Enrichr Appyter.

## 3. Results

### 3.1. ALLO Alters Overall Patterns of Differential Gene Expression in PPD LCLs

Principal Component Analysis showed greater clustering by diagnostic group rather than treatment, suggesting that transcriptomic variance between LCL samples was largely driven by PPD, not ALLO (Figure S1). Replicating previous baseline (i.e., DMSO-treated) findings from whole transcriptome RNA-seq (Rudzinskas et al., 2023), scatter and volcano plots indicated a bias toward downregulated gene expression in PPD compared to Control LCLs (Figure S2A,C). However, this bias toward decreased PPD gene expression disappeared after ALLO-treatment (Figure S2B,D).

### 3.2. Differential GAD1 and Synaptic-Related Gene Network Expression in PPD LCLs

**Figure 2:**
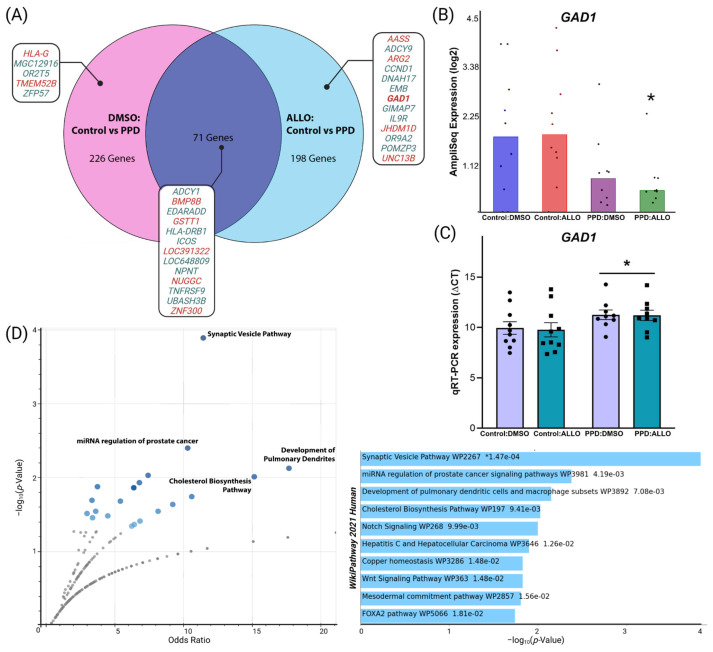
***GAD1* and synaptic signaling-related pathways are differentially expressed in PPD LCLs**. (**A**) Venn Diagram comparing differential gene expression between PPD vs. Control LCLs at (DMSO) baseline compared to after ALLO-treatment. Nominal DEGs highlighted are those with two-fold change or greater, with red indicating decreased expression and green indicating increased expression in PPD relative to controls. (**B**) One of the 13 ALLO-specific DEGs with two-fold change or greater was *GAD1*, a gene for the 67-kDa isoform enzyme GAD67 that catalyzes the conversion of glutamic acid to GABA. Bar chart shows RNA-seq expression data for each sample between all four treatment groups (* = p_nom_ < 0.02, Control:ALLO vs. PPD:ALLO). (**C**) Technical replication via qRT-PCR demonstrated a main effect of Diagnosis (F_(1,34)_ = 5.25, *p* = 0.0283) on *GAD1* expression, which was decreased in PPD LCLs compared to Controls. ALLO-treatment did not show a significant main effect (F_(1,34)_ = 0.0061) or Interaction (F_(1,34)_ = 0.0108, ns). (**D**) Gene Network Analyses of the 198 between-diagnosis DEGs unique to ALLO-treated LCLs were subjected to network analysis via the WikiPathways Human 2021 Database. Top terms are plotted as the Odds Ratio (x-axis) vs. Significance/-log(*p*-Value) (y-axis) for each functional category. Bar chart highlights the top enriched terms, with light blue colored bars corresponding to terms with significant *p*-values (<0.05) and an asterisk (*) indicating a significant adjusted *p*-value.

Transcriptomic analyses revealed 269 significant, differentially expressed genes (DEGs, p_nominal_ < 0.05) between ALLO-treated LCLs (Control:ALLO vs. PPD:ALLO, Table S2), just slightly fewer than the 297 DEGs present at baseline between vehicle-treated LCLs (Control:DMSO vs. PPD:DMSO, Table S1). In addition to the similar number of DEGs between PPD and Controls at baseline and after ALLO-treatment, 71 DEGs were overlapping. Notably, we observed that many non-overlapping DEGs just missed the threshold for significance in either the baseline (e.g., *GAD1*, *UNC13B*) or ALLO comparison (e.g., *IL9R*) (Figure 2A).

Since no DEGs in any comparison with a log(fold change) ≥|1.25| survived false discovery rate (FDR) correction (p_FDR_ < 0.1), only nominally significant DEGs with ≥|2|-fold change (as highlighted in Figure 2A) between Control:ALLO vs. PPD:ALLO were further examined individually. Of the DEGs that met these criteria, the most ostensibly biologically relevant was *GAD1*, the gene encoding the 67-kDa isoform enzyme GAD67 that catalyzes the conversion of glutamic acid to GABA (Figure 2B). Although technical replication via qRT-PCR did confirm that *GAD1* expression was lower in PPD compared with Control LCLs (Diagnosis, F_(1,34)_ = 5.25, *p* = 0.0283), this expression difference was ultimately independent of the presence or absence of ALLO (Treatment, F_(1,34)_ = 0.0061, ns; Interaction, F_(1,34)_ = 0.0108, ns) (Figure 2C).

The unique 198 DEGs identified between Control:ALLO vs. PPD:ALLO LCLs were then subjected to comparative gene enrichment analysis via EnRichr. The WikiPathway Human 2021 database revealed significant enrichment for the synaptic vesicle pathway (*p* < 0.0002, Odds Ratio = 11.13), development of pulmonary dendritic cells and macrophage subsets (*p* < 0.0071, Odds Ratio = 18.36) and the cholesterol biosynthesis pathway (*p* < 0.0094, Odds Ratio = 15.53) (Figure 2D). In comparison, the unique 226 DEGs between Control:DMSO vs. PPD:DMSO LCLs were instead most associated with terms such as ovarian infertility (*p* < 0.0005) and sphingolipid metabolism (*p* < 0.0024) (Figure S3).

### 3.3. PPD LCLs Have Diminished, Divergent Transcriptomic Responses to ALLO Compared with Controls

**Figure 3:**
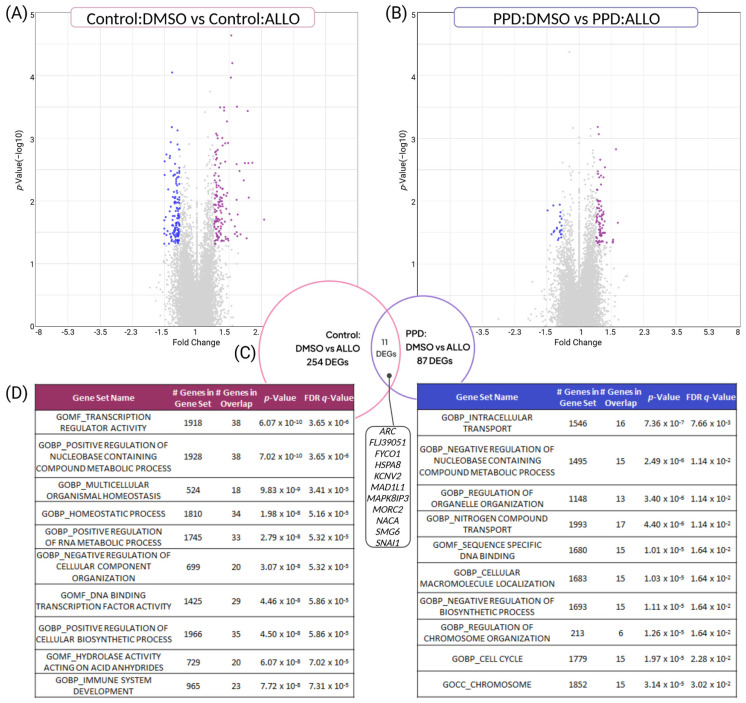
**Unique and opposing ALLO-responsive genes and cellular pathways are induced in PPD LCLs compared with controls**. (**A**,**B**) Volcano plots depicting gene significance (y-axis, (*p* value(−log_10_)) and fold change (x-axis) in transcription after ALLO-exposure for DEGs within Control LCLs and PPD LCLs. Each dot represents one gene. A positive fold change (purple dots, right side of volcano) indicates ALLO increased expression. While Control LCLs had 265 DEGs induced following treatment with ALLO, PPD LCLs exposed to the same ALLO-treatment only had 98 DEGs. (**C**) Venn Diagram comparing DEGs within PPD and Control LCLs after ALLO compared to their respective vehicle (DMSO) baseline. Listed are the 11 nominally significant diagnosis-independent, ALLO-responsive DEGs. (**D**) MSigDB data identifying overrepresented molecular functions in Control-ALLO and PPD-ALLO Gene Ontology. None of the Top 10 most significant ALLO-responsive gene sets overlapped between Control or PPD LCLs. Remarkably, among the top ALLO regulated pathways in both PPD and Control LCLs was regulation of nucleobase containing compound metabolic process. However, this term was positively regulated in Control LCLs, but negatively regulated in PPD LCLs, pointing to a divergent response to ALLO driven by PPD.

ALLO-induced differential expression was then characterized within LCL diagnostic background. Surprisingly, given the potential pathway-relevance of the DEGs identified between groups after ALLO-treatment, substantially more DEGs were induced by ALLO within Control LCLs (Control:DMSO vs. Control:ALLO, 265 DEGs, Table S3), compared to within PPD LCLs (PPD:DMSO vs. PPD:ALLO, 98 DEGs, Table S4). In addition to the differences in quantity, ALLO-induced DEGs in PPD LCLs were prominently upregulated (with 77 of the 98 transcripts increased after ALLO) (Figure 3B and Figure S4B) whereas quantities of up- and down-regulated transcripts were relatively equal in Control LCLs (Figure 3A and Figure S4A). Of the more than 350 total ALLO-induced DEGs, only 11 overlapped between PPD LCLs and Controls (Figure 3C).

Since many different genes can converge to serve similar cellular functions, MSigDB Gene Set Ontology Analysis was used to broadly examine similarities and differences amongst the gene ontologies related to the ALLO-DEGs within each diagnostic group. Highly significant (q-value) gene ontologies corresponding to PPD:DMSO vs. PPD:ALLO DEGs were not only unique from Control:DMSO vs. Control:ALLO DEGs; some networks (e.g., Regulation of Nucleobase Containing Compound Metabolic Process) were directionally (e.g., positive vs. negative) opposite (Figure 3D). Taken together, ALLO-treatment induced both quantitative and qualitative transcriptomic differences in DEGs, ontological networks and cellular responsivity in PPD LCLs compared with Controls.

## 4. Discussion

Allopregnanolone induces a rapid, long-lasting antidepressant response in PPD [7,51]. This response is thought to be linked to the activation of GABA_A_Rs, leading to alterations in synaptic and cellular activity that result in downstream molecular changes [14]. With this in mind, we used our in vitro PPD model to (1) empirically define the transcriptomic consequences of 60 h of ALLO exposure between PPD and Control LCLs, and additionally, (2) evaluate the similarities and differences in cellular response to ALLO within both PPD and Control LCLs. Given the diagnosis-related intrinsic transcriptomic differences previously observed within these LCLs [19], our predictions were that (1) ALLO would mitigate gene expression differences between-diagnosis (i.e., in relation to “baseline” differences), and that (2) although specific ALLO-induced DEGs within-diagnosis would likely diverge, their broader cellular roles would converge.

Our findings were, to some extent, contrary to both predictions. ALLO-treatment did mitigate the proportion of downregulated DEGs in PPD LCLs that has now been repeatedly [19,52,53] observed. However, while 60 h of brexanolone induces a robust, long-lasting therapeutic response in women with PPD, an equally timed in vitro ALLO-exposure only modestly affected overall gene expression compared to PPD diagnosis. Some ~15% of DEGs overlapped in between-group (Control vs. PPD) comparisons (Figure 2A), and this already highly significant percent overlap is most certainly an underestimate due to the limitations of transcriptomics in a relatively small sample size. For example, *GAD1*, a DEG we identified because it met significance criteria in the Control:ALLO vs. PPD:ALLO AmpliSeq comparison, also showed decreased expression that just missed significance (p_nom_ = 0.11) at baseline (Figure 2B). It, therefore, is not represented in this between-diagnosis overlap. However, qRT-PCR replication analyses more concretely demonstrated the significant decrease in *GAD1* expression was driven by a significant main effect of PPD, statistically independent of ALLO-treatment (Figure 2C).

Methodological limitations aside, these findings are consistent with our previous observations of more robust trait- (e.g., PPD vs. Control) versus state- (e.g., exogenous E2 and P4) induced differences in gene expression [19]. However, differences in GABA_A_R subunit expression in LCLs may, in part, also explain ALLO’s seemingly modest effects. On one hand, LCLs are GABA-responsive [36] and express a variety of GABA_A_R subunits [37], including α4, which is highly responsive to ALLO [54]. This literature corroborates the substantial number of DEGs we identified after ALLO-treatment within Control LCLs. However, LCLs do not appear to express the delta subunit, which is prominently expressed in extrasynaptic GABA_A_R that tonically modulate synaptic activity [12,55]. ALLO’s affinity for extrasynaptic GABA_A_Rs has been suggested to be potentially responsible for differences in the therapeutic efficacy of neurosteroids over other classic GABA_A_R modulators, such as benzodiazepines [56], but these interactions are complex. For example, exposure to ALLO can alter GABA_A_R subunit configurations [57] such that subsequent exposures generate anxiety (versus anxiolysis) [58,59], and genetic alterations in GABA_A_Rs may induce paradoxical behavioral responses to ALLO withdrawal [60].

To ultimately define the association (or lack thereof) between ALLO and DEGs such as *GAD1*, larger studies in more complex models, such as induced stem cell-derived neurons, and/or neural tissue from GABA_A_R knockout mouse models of PPD [60] will be necessary. Despite potential limitations of our non-neural PPD LCL model, the gene networks and DEGs identified (such as *GAD1*, an indirect indicator of altered GABA release, and *UNC13B*, a contributor to synaptic vesicle maturation in certain excitatory synapses) tended to functionally correspond to synaptic signaling and neuronal modulation, in support of existing literature linking PPD risk to dysregulated synaptic activity [54,60] (Figure 2D). Moreover, the directionality of expression differences (*GAD1* being downregulated and *UNC13B* being upregulated in PPD) was in line with existing hypotheses that ALLO’s dramatic decline at parturition triggers PPD symptoms by critically decreasing inhibitory signaling, thereby further exacerbating already dysregulated excitatory neurotransmission [14].

Regarding our within-group findings, two primary DEG-related observations were made: PPD LCLs had a diminished transcriptomic response to ALLO (fewer DEGs overall) compared to Controls, and ALLO-responsive PPD DEGs tended to be upregulated (with >75% of PPD having increased expression after ALLO) (Figure 3A–C). These differences also corresponded to a total divergence of significant ALLO-related ontological networks between PPD and Control LCLs (Figure 3D). Although seemingly counterintuitive, intrinsically diminished cellular responsivity in PPD is supported by a growing body of literature, including demonstrations of increased methylation [53,61], diminished neural oscillations [11], aberrant neuroimmune-inhibition [41,42], and blunted cellular stress responses [19,62] in PPD. Additionally, ALLO’s preferential upregulation of transcripts is in line with broader neurophysiologic hypotheses regarding the GABAergic system in PPD [63] and other disorders, such as alcohol use disorder [64].

While longitudinal studies are needed to evaluate whether diminished transcriptomic responses to ALLO are present as cause or consequence of PPD, findings in rodent models investigating the roles of ALLO and early life stress support the former [65]. More broadly, our within-group PPD data also provide in vitro transcriptomic evidence of ALLO’s ability to induce divergent intracellular responses that are dependent on diagnosis (i.e., genetic background) potentially lending support to clinical observations [26], and the hypothesis that ALLO’s actions are highly pleiotropic [63]. Such pleiotropy or differential responses to neurosteroids would have important clinical implications for the wide variety of disorders for which ALLO is currently being evaluated in vivo for therapeutic efficacy, with 14 studies underway and 31 studies listed as completed (ClinicalTrials.gov; accessed on 13 April 2023).

In summary, the striking lack of concordance in DEGs after ALLO-treatment suggests that ALLO mediates unique, and potentially opposing, cellular responses in women with PPD compared with heathy control women. Despite the restrictions of an LCL model that may limit the ultimate generalizability of these findings, these transcriptomic data establish the potential for PPD-specific molecular responses to ALLO to be tied to its antidepressant mechanisms. As such, ALLO’s capacity to play distinct, diagnosis-dependent cellular roles may warrant consideration when evaluating the broader translational and therapeutic potential of this neurosteroid.

## Data Availability

Summary AmpliSeq expression data will ultimately be available in dbGaP. All other data is available upon request.

## Supplementary Materials

The following supporting information can be downloaded at: https://www.mdpi.com/article/10.3390/genes14061234/s1, Figure S1: Unsupervised Clustering of RNA-sequencing; Figure S2: Control vs. PPD, Scatter and Volcano plots; Figure S3: WikiPathway results for 226 DEGs significant only at baseline between PPD and Controls Figure S4: DMSO vs. ALLO (within-diagnosis) Scatterplots; Table S1: Control vs. PPD–Baseline/DMSO (60 hrs Vehicle); Table S2: Control vs. PPD–ALLO (60 hrs ALLO, total 300 nM); Table S3: Control–Baseline/DMSO vs. Control–ALLO; Table S4: PPD–Baseline/DMSO vs. PPD–ALLO.
