# Supplementary material for: Divergent Transcriptomic Effects of Allopregnanolone in Postpartum Depression
Source: Genes (Basel). 2023 Jun 8;14(6):1234. doi: 10.3390/genes14061234 (PMC10298697; doi:10.3390/genes14061234)
Supplement: Supplementary file 1 [file genes-14-01234-s001.zip › PPD_ALLO_SupplementalFiguresTables_05.15.23.pdf]

Supplemental Figures (S1-S4)

Supplemental Table Captions

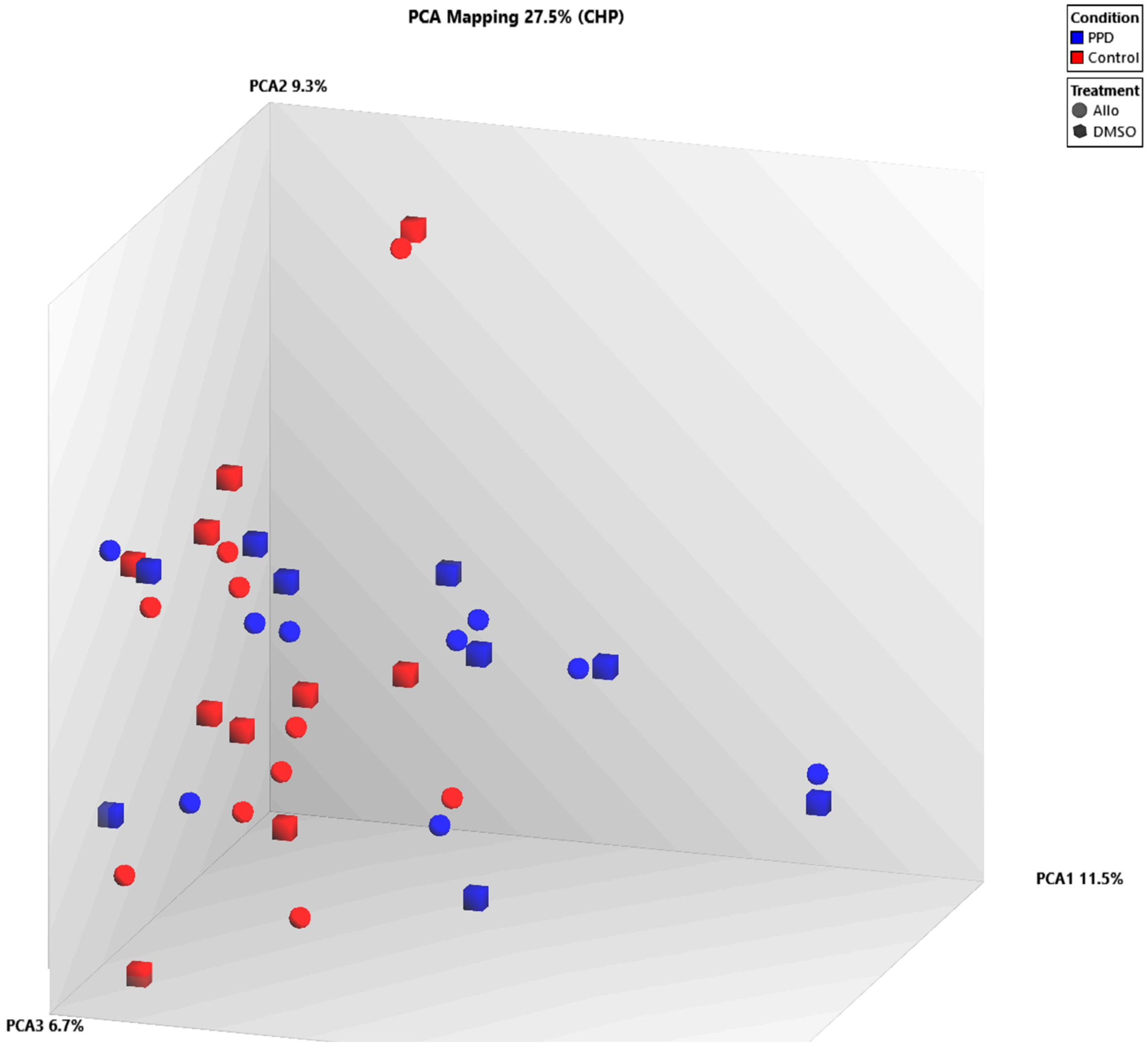

Figure S1: Unsupervised Clustering of RNA-sequencing

A) Principal component analysis (PCA) of the transcriptomic output, using default Transcriptome Analysis Console (TAC) 4.0 settings (5000 points, distributed) to plot all samples in the study. Collectively, the first two axes (PCA 1 & 2) explain 20.8% of the variance, and the top three PCs explaining 27.5% of the variance. Clustering shows segregation of LCL samples primarily between diagnostic groups genetic background, e.g., PPD vs. Control.

Diagnosis (**Color**): Red = Control, and Blue = PPD

Treatment (**Shape**): = DMSO or **O** = ALLO

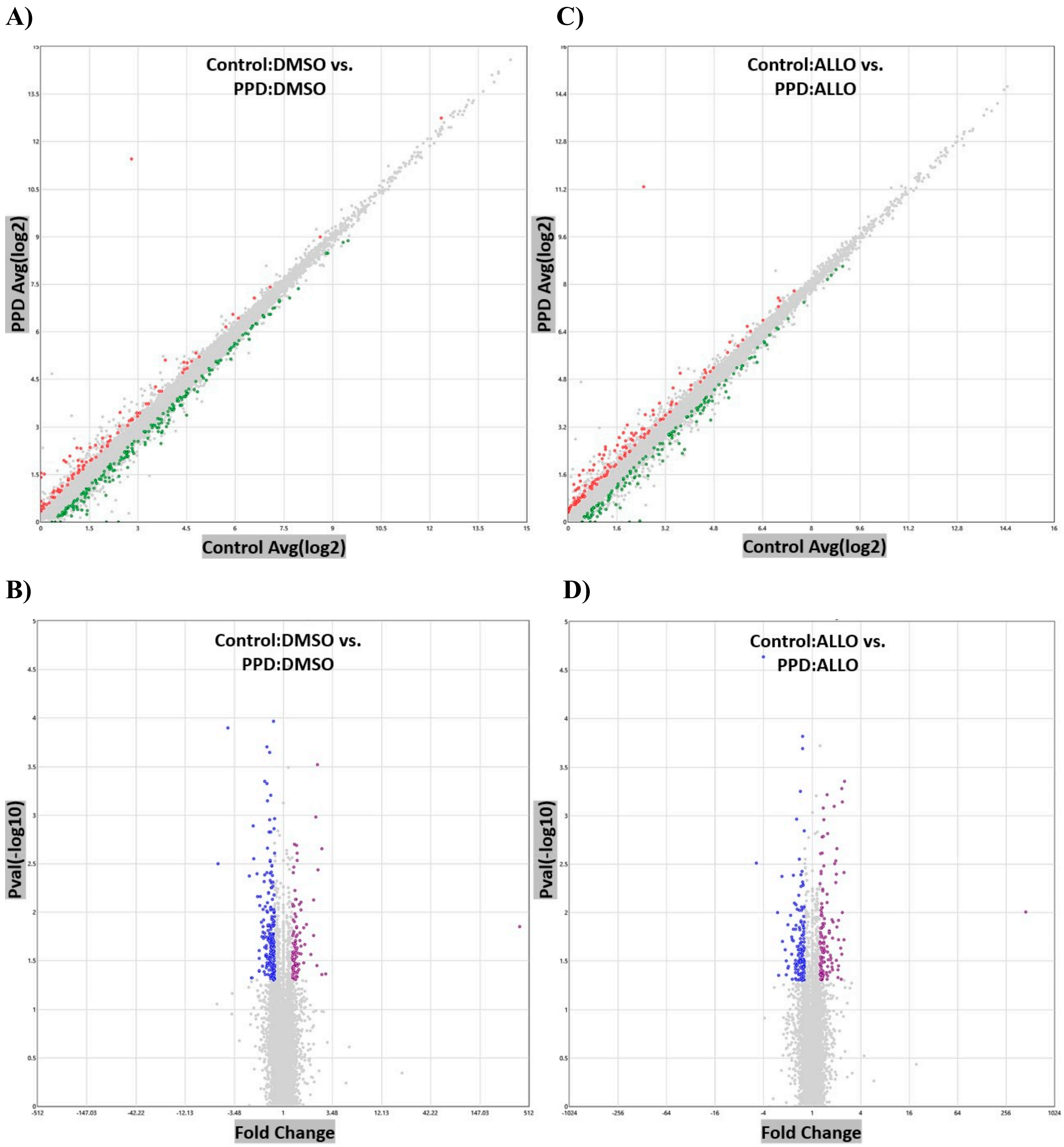

<u>Figure S2: Control vs. PPD, Scatter and Volcano plots</u>

A, C) Scatter plots depicting the transcripts changed in PPD vs. Control LCLs at Baseline and after ALLO-treatment, respectively. The log2(Average Counts) Control is plotted on the X-axis, and log2(Average Counts) PPD is plotted on the Y-axis. Each dot represents one gene.

Red: Upregulated Differentially Expressed Gene (DEG, $p_{nominal}$<0.05) in PPD
Green: Downregulated Differentially Expressed Gene (DEG, $p_{nominal}$<0.05) in PPD

B, D) Volcano plots depicting the nominal significance and fold change in transcription for PPD vs. Control LCLs at Baseline and after ALLO-treatment, respectively. Each dot represents one gene. Fold change is plotted on the x-axis, where a positive fold change (purple, right side of volcano) indicates increased expression in PPD LCLs. The y-axis plots gene significance ((p value)-$\log_{10}$).

Blue = Downregulated Differentially Expressed Gene ($p_{nominal}$<0.05)
Purple = Upregulated Differentially Expressed Gene ($p_{nominal}$<0.05)

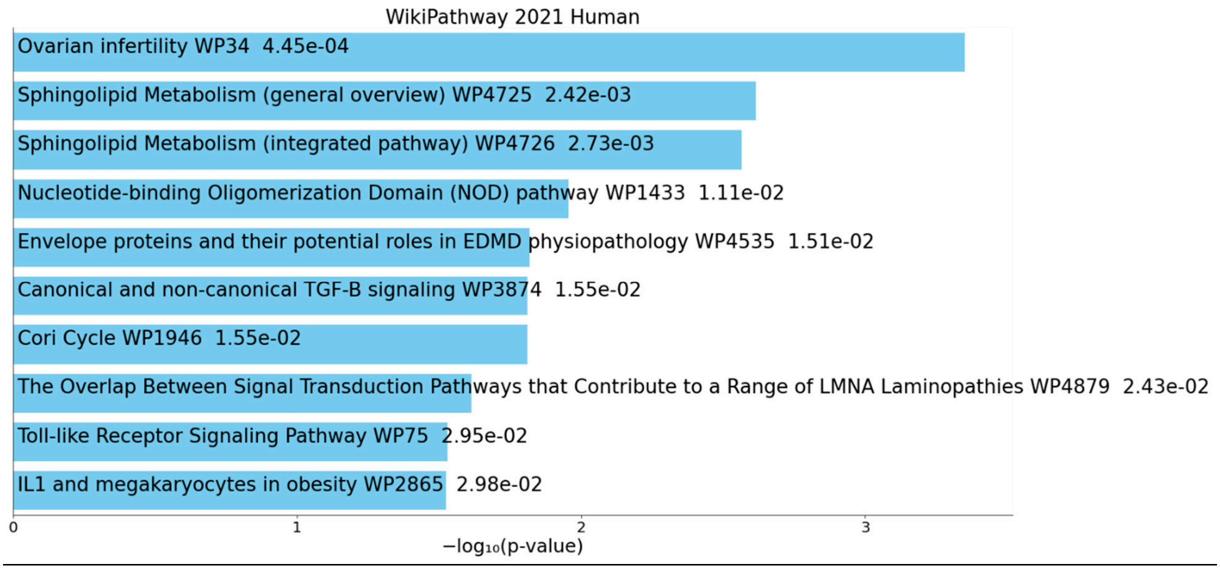

Figure S3: WikiPathway results for 226 DEGs significant only at baseline between PPD and Controls.

Data are plotted as the -log(pValue) for each potential functional category of significance. The bar chart shows the top enriched terms in the chosen database's library, where colored bars correspond to terms with significant p-values (<0.05), and an asterisk (*) indicates the term has a significant adjusted p-value.

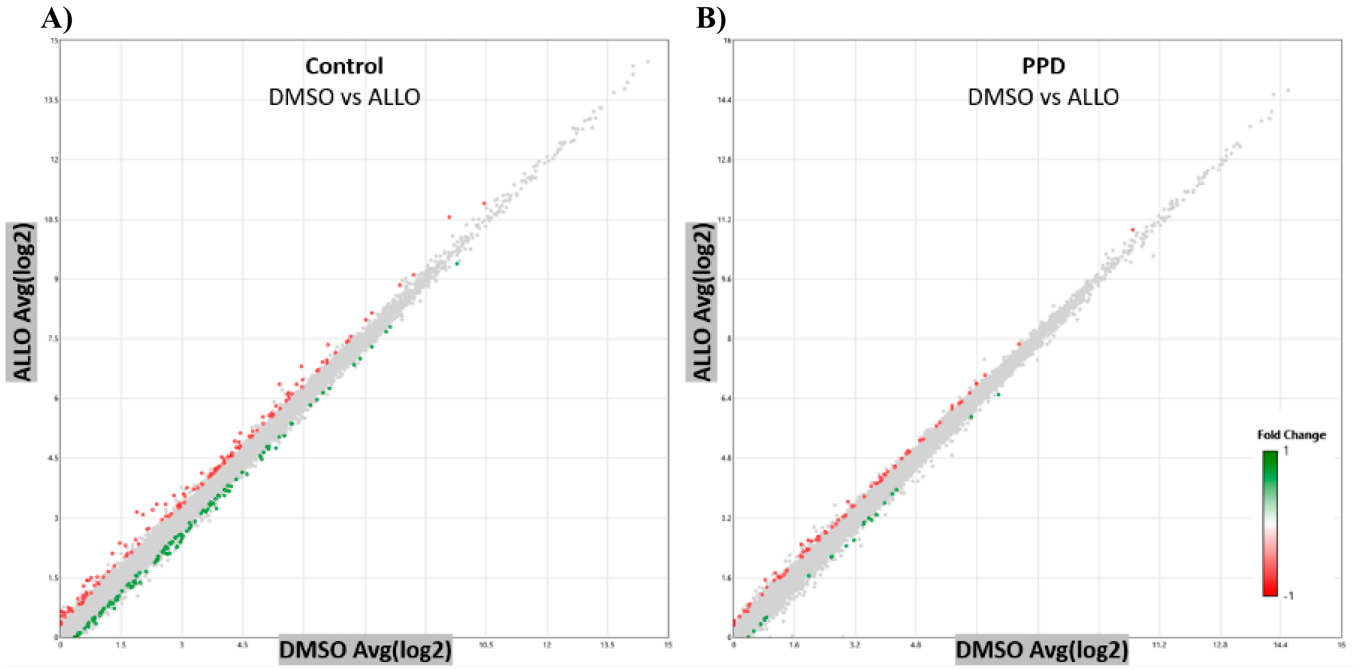

Figure S4: DMSO vs. ALLO (within-diagnosis) Scatterplots

A, B) Scatter plots depicting the transcripts changed after ALLO within Control LCLs and within PPD LCLs, respectively. The log2(Average Counts) DMSO is plotted on the X-axis, and log2(Average Counts) ALLO is plotted on the Y-axis. Each dot represents one gene.

Red: Upregulated Differentially Expressed Gene ($p_{nominal}<0.05$) by ALLO

Green: Downregulated Differentially Expressed Gene ($p_{nominal}<0.05$) by ALLO

Supplemental Tables (Tables are in separate excel sheets):

Table S1,2: RNA-seq results comparing case (PPD) vs. control LCLs within each drug exposure condition

S1: Control vs. PPD – Baseline/DMSO (60hrs Vehicle)

S2: Control vs. PPD – ALLO (60hrs ALLO, total 300nM)

Table S3,4: RNA-seq results comparing LCL baseline (DMSO) to ALLO-treatment within each diagnosis

S3: Control – Baseline/DMSO vs. ALLO

S4: PPD – Baseline/DMSO vs. ALLO
